# Raloxifene Has No Efficacy in Reducing the High Bone Turnover and the Risk of Spontaneous Vertebral Fractures after Denosumab Discontinuation

**DOI:** 10.1155/2018/5432751

**Published:** 2018-09-17

**Authors:** Elena Gonzalez-Rodriguez, Delphine Stoll, Olivier Lamy

**Affiliations:** ^1^Center of Bone Diseases, CHUV, Lausanne University Hospital, Lausanne, Switzerland; ^2^Service of Endocrinology, Diabetes and Metabolism, Lausanne University Hospital, Lausanne, Switzerland; ^3^Service of Internal Medicine, CHUV, Lausanne University Hospital, Lausanne, Switzerland

## Abstract

At denosumab discontinuation, an antiresorptive agent is prescribed to reduce the high bone turnover, the rapid bone loss, and the risk of spontaneous vertebral fractures. We report the case of a woman treated with aromatase inhibitors and denosumab for 5 years. Raloxifene was then prescribed to prevent the rebound effect. Raloxifene was ineffective to reduce the high bone turnover and to avoid spontaneous clinical vertebral fractures. We believe that among the antiresorptive treatments, the most powerful bisphosphonates should be favored, and their administration adapted according to the serial follow-up of bone markers.

## 1. Introduction

Denosumab reduces bone resorption, increases bone mineral density (BMD), and reduces fracture risk [1]. Adjuvant denosumab of 60 mg twice a year reduces the risk of clinical fractures by 50% in postmenopausal women with breast cancer receiving aromatase inhibitors (AI) [2]. Denosumab is widely prescribed in women with postmenopausal osteoporosis, in men at increased risk for osteoporotic fracture, and in patients receiving adjuvant AI therapy for breast cancer or androgen deprivation therapy for prostate cancer at high risk for fracture.

Denosumab discontinuation is associated with a rebound effect which manifests by a severe increase of bone turnover markers and a rapid loss of BMD. Cessation of denosumab after four 60 mg injections induced an increase of C-telopeptide of type I collagen (CTX) above baseline values for two years and a decrease of BMD to the baseline value after one year [3]. In patients who received denosumab for 8 years and did not take any medication for osteoporosis afterward, the mean BMD change over the 1-year observation was −7.4% at the lumbar spine and −7.8% at the total hip [4]. Independent of treatment duration, the increase in BMD at the lumbar spine is thus partially or completely lost within 1 year of denosumab discontinuation. Total hip BMD loss in the year following denosumab discontinuation is equal to or greater than the gain achieved during treatment.

Since 2015, several cases of multiple spontaneous clinical vertebral fractures after denosumab discontinuation were reported and aggregated in a review [5]. These 24 postmenopausal women experienced 112 spontaneous vertebral fractures (mean number per women, 4.7) in the 8 to 16 months (median, 11.2) following last denosumab injection. At the time of fracture diagnosis, CTX values were 2 to 3 times higher than the upper limit of the normal range for premenopausal women [6]. When combining women taking or not taking a medication for osteoporosis after denosumab discontinuation, the risk of clinical vertebral fractures in the 12 months following last denosumab injection is 8.5% [4]. This risk is higher than 10% and probably close to 15% considering a follow-up of 2 years after denosumab discontinuation without taking another osteoporosis treatment [7–9]. Clinical consequences are severe since these fractures are multiple in more than two-thirds of the cases [4, 7]. It is therefore imperative to avoid them. Prescribing a bisphosphonate at denosumab discontinuation would at least reduce bone loss of the lumbar spine [4, 10]. Although there is actually no study showing that such a strategy would prevent the risk of vertebral fractures, several authors and medical societies advocate, at denosumab discontinuation, for a period of treatment with a bisphosphonate or another antiresorptive agent (estrogens or SERMs) to preserve BMD gain and avoid the risk of vertebral fracture [7, 11, 12].

We report the case of a woman who suffered spontaneous clinical vertebral fractures at denosumab discontinuation despite a preventive treatment with raloxifene.

## 2. Case Report

**Figure 1:**
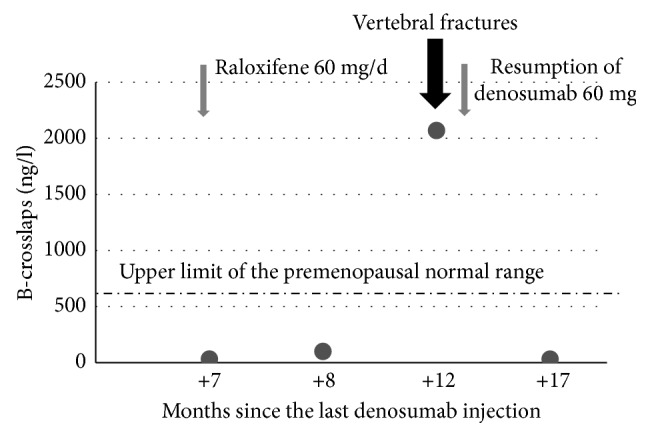
Evolution of B-crosslaps after denosumab discontinuation.

Breast cancer was diagnosed in this 60-year-old woman in July 2010. Initial treatment consisted of surgery, radiotherapy, and chemotherapy. AI therapy with letrozole was started in February 2011 for 5 years. She had no other risk factors for osteoporosis. A DXA performed in March 2011 revealed osteoporosis. BMD T-scores were −2.9 at the lumbar spine and −1.9 at the total hip. Vertebral morphometry confirmed the absence of fractures. The 10-year probability of major osteoporotic fractures assessed by FRAX® was 13%. A treatment with 60 mg denosumab every 6 months and adequate daily calcium and vitamin D supplementation started in March 2011. She received 12 half-yearly injections of denosumab, the last one in August 2016. Letrozole treatment ended in November 2016. A DXA performed in November 2016 showed no more osteoporosis. The lumbar spine and total hip T-score values were −1.7 SD (+18%) and −1.4 SD (+8%), respectively. Vertebral morphometry confirmed the absence of fractures. CTX (fasting blood sample in the early morning, normal range for premenopausal women: 25–573 ng/l) were measured at 33 ng/l in March 2017, 7 months after last denosumab injection. To prevent the high-turnover bone loss associated with denosumab discontinuation, an antiresorptive treatment was proposed. She refused bisphosphonates for fear of side effects. Raloxifene 60 mg daily was accepted and started in March 2017. In April 2017, CTX values were low at 100 ng/l. The patient scrupulously took her treatment. By mid-July, she experienced spontaneous low back pain. Thoracolumbar MRI performed in August revealed two D11 and L5 fractures with medullary edema. CTX, measured in August 2017, were extremely high at 2070 ng/l (Figure 1). To rapidly reduce the increased bone turnover, an injection of denosumab was given at the time of fracture diagnosis.

## 3. Discussion

This case report illustrates the difficulty of managing denosumab discontinuation and raises several questions. Raloxifene has not been effective, neither in reducing the high bone turnover nor in preventing spontaneous vertebral fractures. In addition, the interval between consecutive measurements of CTX was too long. It did not allow us to detect early the increase of bone turnover markers and to adapt antiresorptive treatment.

Some case reports suggest that the rebound effect is reduced in patients treated with bisphosphonates after denosumab discontinuation or before denosumab initiation [4, 10, 13]. The antiresorptive effect of raloxifene is probably not powerful enough to counter the severity of the rebound effect associated with denosumab discontinuation. One way to quantify the antiresorptive effect of osteoporosis treatments is to measure the decrease in markers of bone turnover in patients treated for osteoporosis. In the MORE trial, raloxifene 60 mg daily decreases the bone turnover markers by 26.2% to 35.2% [14]. Bisphosphonates are more powerful antiresorptive agents than SERMs, but their antiresorptive potency varies from agent to agent. Oral daily or quarterly intravenous injection of ibandronate decreases serum CTX by 53.4% to 59.9% [15]. In a 12-month head-to-head trial, once-weekly alendronate 70 mg was more efficacious than once-weekly risedronate 35 mg to decrease serum CTX (73.8% vs. 54.7%) [16]. Intravenous zoledronate 4 mg decreases serum CTX at one month by 83% and at 1 year by 52% [17]. In order to minimize the high bone turnover at denosumab discontinuation, it seems therefore preferable to prescribe alendronate or zoledronate. In our case report, raloxifene had strictly no efficacy in reducing the high bone turnover. However, the choice of a powerful bisphosphonate does not guarantee sufficient inhibition of the high bone turnover markers increase and secondary bone loss [10]. Thus, for alendronate or zoledronate, it may be necessary to administer them either at closer frequencies or at higher doses to achieve the desired effect.

Moreover, since bisphosphonates are deposited on areas of bone resorption, they must probably be administered when the rebound effect associated with the denosumab discontinuation has already begun as measured by the increase in bone turnover markers. This consideration is of little importance if an oral bisphosphonate is administered repeatedly but may be essential if one course of zoledronate is administered [10, 18]. Delaying administration of intravenous bisphosphonate when transitioning from denosumab was demonstrated to maintain the gains in BMD [19]. Repeated measurements of serum CTX, every month or two months for at least six months since the theoretical end of denosumab effect, would therefore be imperative in this situation. Indeed, it seems that denosumab efficacy duration is different between patients, and increase in turnover markers develops very quickly once started. In our patient, CTX were very low 7 months after last denosumab injection (33 ng/l). The slight increase measured one month later was interpreted as a controlled rebound effect by raloxifene (100 ng/l). It was however the beginning of the significant increase observed later. Frequent measurements of bone turnover should make possible: (1) to detect the beginning of the rebound effect associated with denosumab discontinuation; (2) to evaluate the effectiveness of the given antiresorptive treatment; and, if necessary, (3) to replace it or to adjust its dosage. However, the threshold value that determines the need for an intervention is yet unknown. Moreover, suppression of bone resorption is currently not proven to prevent bone loss and to avoid the risk of vertebral fractures.

A prior exposure to bisphosphonates before starting denosumab may be another strategy to reduce the high bone turnover when discontinuing denosumab therapy [13]. As bisphosphonates are incorporated into the bone matrix, their inhibitory effectiveness on bone turnover may persist several years after the end of therapy. This strategy seems not to be valid for the prevention of spontaneous vertebral fractures after denosumab discontinuation. Nine women with prolonged exposure to bisphosphonates prior denosumab initiation experienced 36 spontaneous vertebral fractures after denosumab discontinuation [20].

If vertebral fractures occur after denosumab discontinuation, it is urgent to block the high bone turnover. As denosumab is the only antiresorptive treatment that can inhibit bone resorption in a matter of days, it is a possibility to quickly give a new injection of denosumab. Denosumab 60 mg decreases serum B-crosslaps by 83.6% after 3 days [21]. This strategy is simple, but delays the management of denosumab withdrawal for several months or years.

We conclude that, in this patient, raloxifene had no efficacy in reducing the high bone turnover and the risk of spontaneous vertebral fractures after denosumab discontinuation. Powerful bisphosphonates are probably the treatment of choice for men and women who discontinue denosumab. Studies are urgently needed to assess the efficacy of bisphosphonates in such situations.
